# Investigation of a Reduction in Tylosin on the Prevalence of Liver Abscesses and Antimicrobial Resistance in Enterococci in Feedlot Cattle

**DOI:** 10.3389/fvets.2020.00090

**Published:** 2020-02-28

**Authors:** Taylor Davedow, Claudia Narvaez-Bravo, Rahat Zaheer, Haley Sanderson, Argenis Rodas-Gonzalez, Cassidy Klima, Calvin W. Booker, Sherry J. Hannon, Ana L. Bras, Sheryl Gow, Tim McAllister

**Affiliations:** ^1^Food and Human Nutritional Sciences Department, University of Manitoba, Winnipeg, MB, Canada; ^2^Lethbridge Research and Development Centre, Agriculture and Agri-Food Canada, Lethbridge, AB, Canada; ^3^Feedlot Health Management Services, Okotoks, AB, Canada; ^4^Public Health Agency of Canada, Saskatoon, SK, Canada

**Keywords:** Enterococci, antimicrobial resistance, tylosin, erythromycin, tetracycline, beef cattle

## Abstract

Recent concerns over linkages between antimicrobial resistance in human pathogens and antimicrobial use in livestock have prompted researchers to investigate management strategies that reduce the current reliance on in-feed tylosin to control liver abscesses in feedlot cattle. A total of 7,576 crossbred yearlings were allocated to the study (~253 animals/pen, 10 replicate pens per treatment) and individually randomized to one of three treatments. Tylosin phosphate (11 ppm) was included in-feed (1) for the first 125 days on feed (DOF) (**FIRST-78%**), (2) for DOF 41 to 161 (**LAST-75%**), or (3) for the entire feeding period (**CON**; day 0–161). Fecal composites were collected from the pen floor on days 0, 81, and 160 of the finishing period. Serial dilutions were spread plated for enumeration of enterococci on Bile Esculin Azide (BEA) agar and BEA amended with 8 μg/ml erythromycin. Results indicated that although the proportion of Ery^R^ enterococci increased with DOF (*P* < 0.01), neither treatment (*P* = 0.34) or treatment × DOF (*P* = 0.37) affected antimicrobial resistance. Of the 538 isolates, 97% were enterococci, with mixed species isolated early in the feeding period and only *Enterococcus hirae* isolated at the end. Isolates were most frequently resistant to tylosin (86%), erythromycin (84%), and doxycycline (31%). Macrolide and tetracycline resistant isolates harbored *erm*(B), *msr*C, and *tet*(L), *tet*(M), *tet*(O) genes, respectively. Overall, the proportion of Ery^R^ enterococci increased (*P* < 0.05) in all three treatments over the feeding period. Compared to the control cattle, **FIRST-78%** cattle had more severe (*P* < 0.05) liver abscesses, while there was a trend (*P* < 0.08) for this response in **LAST-75%** cattle. There was no difference (*P* > 0.05) in total liver abscesses, growth performance, carcass traits, morbidity, or mortality among treatments. These results support the potential to reduce the duration and therefore quantity of tylosin administered to feedlot cattle during the feeding period without impacting animal productivity.

## Introduction

Liver abscesses have a major economic impact on the North American beef cattle industry, with an average prevalence in feedlot cattle ranging from 12 to 32% (1), but it has been reported to be as high as 95% (2). Cattle with severely abscessed livers can exhibit compromised growth performance as a result of reduced feed intake and carcass weight (3, 4). In Canada, economic losses as a result of condemned and discounted livers are estimated at $60 million annually (5).

Antimicrobials are the primary tool used to prevent liver abscesses in cattle fed high-grain finishing diets. The macrolide, tylosin phosphate, is the most common antimicrobial included in feed to control liver abscess in beef cattle in North America (6), as it targets the causative agents, *Fusobacterium necrophorum* and *Trueperella pyogenes* (7). However, despite its use, the prevalence of liver abscesses in slaughter cattle still often exceeds 15% (5).

The use of antimicrobials in-feed has come under scrutiny by both the public and regulators over concerns that their use selects for antimicrobial resistance and poses a risk to public health (8). Tylosin belongs to the MLS_B_ superfamily (macrolide-lincosamide-streptogramin B) which are classified as a category II antimicrobial in terms of their importance for use in human medicine (9). Although tylosin is not used in human medicine, it cross-selects for resistance to other antimicrobials within this superfamily, including erythromycin, a macrolide widely used in humans (10).

It is essential to evaluate new strategies to manage liver abscesses in feedlot cattle while reducing reliance on medically important antimicrobials in livestock production. According to recently implemented restrictions in the United States (11) and Canada (12), all medically important antimicrobials require a veterinary prescription and cannot be used for growth promotion.

Enterococci are commensal bacteria of humans and animals that are often associated with serious hospital acquired infections (13). The most prevalent species associated with infections in human are *E. faecium* and *E. faecalis* (14), whereas *E. hirae* is the predominant species in cattle (15). Few studies have investigated the link between tylosin administration and antimicrobial resistance in enterococci in cattle. The most recent study in Canada, withdrew tylosin 28 days prior to slaughter in a small-scale (100 steers) trial and found a reduction in macrolide resistance in enterococci (16). Another feedlot study in the United States investigated the impact of intermittent (1 week on, 2 weeks off) and continuous administration vs. no tylosin on erythromycin resistance (Ery^R^) in enterococci. They found no difference in the occurrence of liver abscesses between intermittent and continuous treatments, but there were more liver abscesses in cattle that did not receive tylosin (17). As such, it is important to continue to investigate ways to optimize tylosin use while promoting antimicrobial stewardship, supporting productivity, and working to minimize use of antimicrobials in livestock that are of importance in human medicine.

The present study investigated and compared the effect of tylosin administration in the first 78 or last 75% of the feeding period on antimicrobial resistance, liver abscess score, animal health, feedlot performance, and carcass traits of feedlot cattle.

## Materials and Methods

All procedures involving cattle were reviewed and approved by the Feedlot Health Management Services Ltd. (Okotoks, Alberta) and Lethbridge Research Center Animal Care Committees in accordance with guidelines of the Canadian Council on Animal Care (18). Informed consent for use of the cattle was received from the owners of the cattle.

### Experimental Design

This study was conducted at a large commercial feedlot in southern Alberta over an 161-day finishing period. Cattle (*n* = 7,576) for this study were crossbred beef yearling steers and heifers (394 ± 5.49 kg) that arrived between June 11, 2018 and July 7, 2018. Upon arrival, cattle were randomly assigned to one of three treatments; **FIRST-78%**, **LAST-75%**, or **CON**. The experimental unit was the pen, with 10 pens (six steer, four heifer) allocated to each treatment. Average pen capacity was 253 ranging from 246 to 280 head/pen. Upon arrival, individual animals were managed as per standardized commercial Canadian feedlot practices, receiving an ear tag for identification, a hormonal growth promoter implant, a parenteral respiratory vaccine, a parenteral clostridial disease bacterin, and topical parasite control. No antimicrobials were administered to the cattle upon arrival. Cattle were randomly assigned to one of the three treatments and placed into a corresponding pen. Once a pen was full, then newly arrived cattle were allocated to a new pen for a second replicate of that treatment with this process continuing until all 10 pens per treatment were full.

Cattle were fed tylosin phosphate (Tylosin 40, Bio Agri Mix LP, Mitchell, ON) at an inclusion level of 11 ppm [100% dry matter basis [DM]] for: (1) the first 125 days of the 161-days feeding period (**FIRST-78%**), (2) the last 120 days of the feeding period (**LAST-75%)**, starting at an average of 41 days on feed (DOF) and continuing to slaughter at an average of 161 DOF, or (3) continuously throughout the 161-days feeding period (**CON**). Tylosin was administered at the concentration approved for the prevention of liver abscesses in beef cattle in Canada (19).

All diets were fed twice daily, and cattle were offered *ad libitum* access to feed and water. Using a series of four step-up diets, cattle were gradually transitioned to a high-concentrate finishing diet (dry matter basis) consisting of 85.8% concentrate, 11.5% roughage, and 2.8% supplement. The concentrate portion consisted of 70% corn with the remainder being tempered rolled barley / wheat. Monensin sodium was also included in diets at 33 ppm DM over the feeding period (Monensin Premix; Bio-Agri Mix LP, Mitchell, Ontario) according to the medicating ingredient brochure (19).

### Sample Collection and Processing

Composite, fresh, pen-floor fecal samples from 20 different pats were collected from each pen using a standardized pen sampling plan. Samples were collected at allocation (0 DOF) before any tylosin was administered, in the middle of the feeding period (avg. 81 DOF), and just prior to shipment for slaughter (avg. 160 DOF). Samples were collected in sterile Whirl Pak bags and stored at 4°C for an average of 1 day prior to transport to the Agriculture and Agri-Food Canada Lethbridge Research Center, Lethbridge, Alberta for microbial analysis. Samples were processed within 1 day of arrival at Lethbridge.

At the lab, each fecal sample was thoroughly mixed, weighed (1.0 g) and diluted 1:5 into 4.0 mL of sterile phosphate buffered saline and vortexed for 30 s. Samples were then 10-fold serially diluted and 100 μL of the appropriate dilution were plated in duplicate onto Bile Esculin Azide (BEA) agar containing no antimicrobials and BEA amended with erythromycin (8 μL/mL; BEA^E^). The concentration of erythromycin added into the BEA plates was set at the breakpoint standards for defining resistance as described by the Clinical and Laboratory Standards Institute (CLSI) guidelines (20). After incubation for 48 h at 37°C, colonies that exhibited esculin hydrolysis (black precipitate) and morphology typical of enterococci were enumerated. Isolates that grew on BEA^E^ were considered resistant to erythromycin. The percentage of enterococci resistant to erythromycin was calculated according to Alexander et al. (21), in which: [(number of colonies on selective BEA^E^ plates / total colonies on non-selective BEA plates) × 100%].

For each sample, three enterococci colonies each from BEA and BEA^E^ plates (6 colonies in total) were subcultured onto their respective media and incubated for 48 h at 37°C, for purification and further characterization. To prepare template DNA for PCR, one colony from each plate was suspended in 100 μL of TE (10 mM Tris, 1 mM EDTA, pH 8.0) and heat lysed for 5 min at 98°C with shaking at 1000 RPM in an Eppendorf thermomixer (VWR, Missisauga, ON). Heat lysed cell suspensions were stored at −80°C for later use. Growth from subcultures was suspended in brain heart infusion (BHI) broth containing 15% glycerol and archived at −80°C for subsequent use.

### Characterization of *Enterococcus* Species

A total of 538 presumptive enterococci isolates representing approximately six isolates from each pen on each sampling day were saved in TE as mentioned above. Tubes containing heat lysed cells were thawed and centrifuged at 10,000 × g for 5 min. The supernatant was used as the template DNA in a multiplex PCR to identify *Enterococcus* species. *Enterococcus*-specific *groES-EL* primers Ent-ES-211-233-F and Ent-EL-74-95-R (22) were used along with *Enterococcus hirae* muramidase gene (23) *mur2*-specific mur2h_F1 (5′-TATGGATACACTCGAATATCTT-3′) and mur2h_R (5′-ATTATTCCATTCGATTAACTGC-3′) primers were used in a multiplex PCR assay to distinguish *E. hirae* from other *Enterococcus* spp. Two microliters of template DNA was used in a 25 μL PCR reaction volume using HotStarTaq Master Mix Kit (Qiagen Canada, Inc., Mississauga, ON, Canada) as per manufacturer's instructions and with the following thermocycler conditions: 5 min at 95°C, followed by 45 cycles of 30 s at 94°C, 30 s at 49°C, 30 s at 72°C and a final extension for 10 min at 72°C. The PCR products were resolved on a 1.8% agarose gel. Isolates that were positive for both primer sets generated two PCR product bands and were identified as *E. hirae*, while single PCR products presumably originating from *groES-EL* positive, but *mur-2* negative (non-*E. hirae*) enterococci isolates were sent to Eurofins Genomics (Toronto, ON) for Sanger sequencing of the *groES-EL* intergenic region to identify species.

### Antimicrobial Susceptibility Testing

A subset of 176 speciated isolates were randomly chosen to represent one isolate from each media type from all samples, with the exception of four isolates from the BEA plates that were not enterococci. Antimicrobial susceptibility testing for enterococci was performed against 12 antibiotics using disc diffusion methodology according to the CLSI guidelines for *Enterococcus* spp., documents M02-A12, M100-S26, and VET-01S (20, 24, 25). The panel covers medically important antibiotics that are classified as either medium, high or very high importance in human medicine (9). The antimicrobial panel, supplier, disk content, and zone diameter for determining break points are listed in Supplementary Table 1. *Staphylococcus aureus* ATCC® 25923 and *Enterococcus faecalis* ATCC® 29212 were used as standards and were included in each assay. Zone diameters were read using the BioMic V3 imaging system (Giles Scientific, Inc., Santa Barbara, CA, USA), and each enterococci isolate was classified as either susceptible, intermediate or resistant according to CLSI guidelines for 10 antimicrobials, or EUCAST for tigecycline (26). Tylosin does not have established interpretive criteria for *Enterococcus* spp., although there is an acceptable quality control range for 30 μg tylosin discs for *S. aureus* ATCC® 25923 set at 18–26 mm (24). For tylosin, previously published minimum inhibitory concentration (MIC) established in our lab (16) were used as breakpoints in the current study. Isolates that were resistant to three or more antimicrobials were defined as multidrug resistant.

### Resistant Gene Determinants

The isolates displaying intermediate resistance or resistance to erythromycin or tylosin were screened by PCR for macrolide resistance genes *erm*(B), and *msr*C (27), using the primers of Chen et al. (28), and Beukers et al. (16), respectively. Reactions were processed as a multiplex PCR with an initial denaturation for 5 min at 95°C, followed by 35 cycles of denaturation for 30 s at 94°C, annealing for 30 s at 58°C and a final extension for 10 min at 72°C. Isolates displaying intermediate resistance or resistance to doxycycline were also screened by PCR for *tet*(L), *tet*(M), and *tet*(O) as previously described (29). All PCRs were prepared as a 20 μL reaction with 2 μL DNA template and resolved on a 1.5% agarose gel. Conventional PCR was performed using HotStarTaq Master Mix Kit, and multiplex reactions using the Mulitplex Master Mix Kit (Qiagen Canada, Inc., Mississauga, ON).

### Animal Performance, Liver Abscesses, and Carcass Traits

Upon allocation, initial body weight (BW) and hip height were measured as baseline variables for each individual animal to assess homogeneity across treatments. Animal performance variables (final BW; daily dry matter intake, DDMI; average daily gain, ADG; feed-to-gain ratio, F:G) were calculated for each pen to describe feedlot performance. Final BW represented the average net (shrink accounted for gut fill) live weight of cattle sold for slaughter. The DDMI was calculated by the total quantity of feed consumed divided by the number of days on feed and animals within a pen. The ADG was determined by the total net slaughter weight plus total weight of cattle shipped for salvage slaughter plus total weight of animals that died minus total allocation weight; divided by the number of days in the trial. Feed efficiency (F:G) was determined as DDMI divided by ADG on a live weight basis. Cattle were monitored twice daily by animal health personnel for evidence of disease. Individual cattle that were deemed “sick” were separated out of the pen and moved to a hospital facility for diagnosis and treatment. If cattle were housed in hospital pens, the feed was accounted for by proration to the home pen record as per standard procedures. An effort was made to avoid treating “sick” cattle with macrolides and they were returned directly to their home pen whenever possible. When this was not possible, their removal from the home pen was accounted for. Overall mortality was defined as the number of mortalities divided by the number of animals allocated.

All animals from this study were slaughtered at a single processing plant. Cattle from assigned pens were shipped for slaughter as a single lot as per finishing time as assessed by standard feedlot production practices. At slaughter, all livers were scored for severity and prevalence of liver abscesses by trained personnel, using a modified Elanco Liver Check System (Elanco, Greenfield, IN, USA). Livers that had no abscesses (normal healthy liver) were assigned a liver score of 0. Livers with one or two small active abscesses/scars or up to four abscesses with a diameter of < 2.5 cm were assigned a liver score of A. Livers with one or more large abscesses (diameter > 2.5 cm) or more than four small/old abscesses of a diameter < 2.5 cm were assigned a liver score of A+ (severe).

Canadian quality grade (QG), yield grade (YG), and weight of each carcass were collected using the data capture system at the processing plant. The average carcass weight was determined by the total carcass weight at slaughter divided by the number of cattle sold for slaughter. The dressing percentage was calculated by the total carcass weight at slaughter divided by the total weight at slaughter expressed as a percentage.

### Statistical Analysis

Data were analyzed using SAS® for Windows, Release 9.4 (SAS Institute Inc., Cary, North Carolina). Prior to analysis, microbial enumeration data were normalized by a log_10_ transformation and analyzed using the MIXED procedure of SAS with a completely randomized factorial arrangement with repeated measures. The treatments (**FIRST-78%**, **LAST-75%**, **CON**) and sampling days (0, 81, 160) and their interaction were analyzed as fixed effects with replicate as a random effect.

The baseline (initial BW and hip height), liver abscess score, feedlot performance, and carcass trait variables were analyzed using GLIMMIX in SAS. Baseline variables were tested as covariates of the feedlot performance variables and included in the model if statistically significant. Sex (steers or heifers) was included as a fixed effect in the models for feedlot performance and liver abscess score. Morbidity and mortality data were analyzed using the GENMOD procedure in SAS with Poisson regression in a log linear model for treatment effects and adjusted for clustering of disease (pen nested within replicate) with generalized estimating equations. For all tests, level of significance was set at *P* < 0.05.

## Results

### CFU Counts of Enterococci and Proportion of Erythromycin Resistance

**Figure 1:**
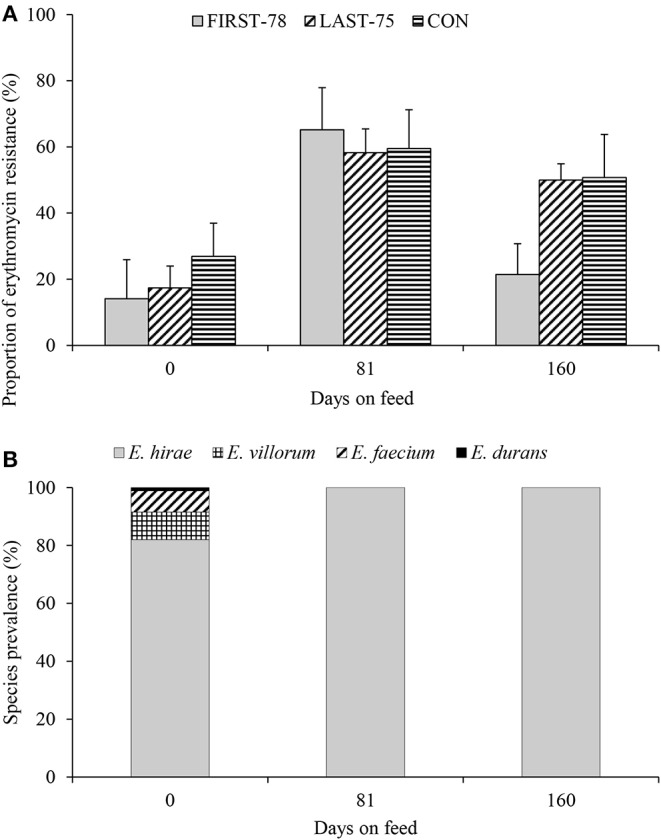
Erythromycin resistance **(A)** and species prevalence **(B)** of enterococci isolated from the feces (sample 0, 81, 160 days) of feedlot cattle administered tylosin in the **FIRST-78%**, **LAST-75%**, or continuously (**CON**) during a 160 days feeding period. **(A)** Treatment group, *P* = 0.34; Treatment group X Days on feed, *P* = 0.37; Days on feed, *P* < 0.01. **(B)** Isolates are pooled across all pens, treatment groups and media type.

Enterococci were isolated from fecal composite samples from all 30 pens on all sampling days with the exception of four pens on day 81, where selected colonies were not enterococci. No difference (*P* > 0.05) was observed between **FIRST-78%**, **LAST-75%**, and **CON** cattle with regard to total enterococci, Ery^R^ enterococci (Table 1), or proportion of Ery^R^ enterococci within the total enterococci population (Figure 1A). However, there was a decrease (*P* < 0.01) in total enterococci with increasing days on feed. The proportion of Ery^R^ was highest on day 81 (*P* < 0.01) for all treatments. Compared to arrival, the proportion of Ery^R^ enterococci isolated just prior to slaughter increased by 52, 187, and 89% (*P* < 0.01) in the **FIRST-78%**, **LAST-75%**, and **CON**, respectively (Figure 1A).

**Table 1:** Enterococci counts of the total population and Ery^R^ *enterococci* isolated from feedlot cattle feces from cattle fed tylosin for the **FIRST-78%**, **LAST-75%**, or continuously (**CON**) during the feeding period.

**Item^**a**^**	**Treatments**^**b**^			**SEM**	***P–*****value**^**c**^		
	**FIRST-78**	**LAST-75**	**CON**		**T**	**D**	**T × D**
**No. of Enterococci (log**_**10**_ **CFU/g feces)**							
Day 0	6.0	6.5	6.2	0.24	0.14	<0.01	0.12
Day 81	5.2	5.7	5.9				
Day 160	5.3	5.3	5.3				
**No. of Ery**^**R**^ **Enterococci (log**_**10**_ **CFU/g feces)**							
Day 0	4.4	4.5	4.9	0.41	0.18	0.02	0.98
Day 81	5.0	5.4	5.4				
Day 160	4.5	4.7	4.9				

a *Cattle were sampled upon arrival and after 81 and 160 days on feed. Total enterococci were enumerated on BEA, bile esculin azide agar; and erythromycin resistant (Ery^R^) enterococci were enumerated on BEA^E^ amended with erythromycin (8 μg/ml)*.

b *Tylosin inclusion at 11 ppm; FIRST-78% = tylosin in-feed from 0 to 125 days; LAST-75% = tylosin in-feed from d 41 to d 161; CON, control, continuous feeding of tylosin (0–161 days)*.

c *T, Treatment; D, Days on feed; T × D, Treatment × Days on feed*.

### Characterization of Enterococci

Of the 538 isolates collected throughout the trial, 97% were confirmed as enterococci by PCR. Speciation of 522 enterococci isolates revealed that 93.9% were *E. hirae* (*n* = 490), 3.3% were *E. villorum* (*n* = 17), 2.5% were *E. faecium* (*n* = 13), and 0.4% were *E. durans* (*n* = 2). Out of the 32 non-*hirae* enterococci isolated, 41% (*n* = 13) were collected from non-selective BEA, whereas 59% (*n* = 19) were isolated from selective BEA^E^. The diversity of enterococci tended to be greater at arrival than later during the feeding period (Figure 1B), with *E. hirae* being the only species identified on day 81 and 160.

### Antimicrobial Susceptibility Testing

Across all treatments, a total of 86% (*n* = 151), 84% (*n* = 147), and 31% (*n* = 54) of isolates displayed intermediate resistance or resistance to tylosin, erythromycin and doxycycline, respectively (Table 2). Ninety-five percent of the isolates (*n* = 145/153) that were not susceptible to macrolides displayed either intermediate resistance or resistance to both erythromycin and tylosin. In total, 16 antibiogram phenotypes were observed, ranging from no resistance (A1) to resistance to six antimicrobials (A16) (Table 2). No isolates displayed intermediate resistance or resistance to ampicillin, gentamicin, levofloxacin, or vancomycin; but at least one isolate was resistant to each of the other antimicrobials tested. The three most common antimicrobial resistance phenotypes across all treatments and days were A1 (No resistance), A5 (ERY-TYL), and A7(dox-ERY-TYL), representing 82% of all observed susceptibility patterns. Multidrug resistance (≥ 3 antimicrobials) occurred in 9.7% (*n* = 17) of isolates, and did not appear to be influenced by treatment.

**Table 2:** Antibiograms of enterococci (*n* = 176) isolated from feedlot cattle feces from cattle fed tylosin for the **FIRST-78%**, **LAST-75%**, or continuously (**CON**) during the feeding period.

**Profile**	**Phenotype^**c**^**	**No. isolates (%) within treatments and days**^a^^,^ ^b^									**Total**
		**FIRST-78**			**LAST-75**			**CON**			
		**d 0** **(*n* = 20)**	**d 81** **(*n* = 20)**	**d 160** **(*n* = 20)**	**d 0** **(*n* = 20)**	**d 81** **(*n* = 18)**	**d 160** **(*n* = 20)**	**d 0** **(*n* = 20)**	**d 81** **(*n* = 18)**	**d 160** **(*n* = 20)**	
A1	No Resistance	6 (30.0)		2 (10.0)	6 (30.0)	1 (5.6)		5 (25.0)	1 (5.6)		21
A2	NIT			1 (5.0)							1
A3	Tyl						1 (5.0)		1 (5.6)	1 (5.0)	3
A4	ery-nit	1 (5.0)									1
A5	ERY-TYL	7 (35.0)	16 (80.0)	4 (20.0)	7 (35.0)	7 (38.9)	10 (50.0)	7 (35.0)	12 (66.7)	11 (55.0)	81
A6	nit-tyl						1 (5.0)	1 (5.0)			2
A7	dox-ERY-TYL	2 (10.0)	3 (15.0)	9 (45.0)	1 (5.0)	10 (55.6)	5 (25.0)	3 (15.0)	4 (22.2)	5 (25.0)	42
A8	ery-lin-NIT				1 (5.0)						1
A9	ERY-nit-TYL		1 (5.0)	1 (5.0)	1 (5.0)		1 (5.0)	1 (5.0)			5
A10	ERY-q-d-TYL	1 (5.0)		2 (10.0)			1 (5.0)				4
A11	ERY-str-TYL									1 (5.0)	1
A12	lin-NIT-TYL	1 (5.0)									1
A13	DOX-ERY-NIT-TYL	2 (10.0)		1 (5.0)	2 (10.0)		1 (5.0)	2 (10.0)		2 (10.0)	10
A14	ery-NIT-TIG-tyl							1 (5.0)			1
A15	dox-ERY-NIT-q-d-TYL				1 (5.0)						1
A16	dox-ery-lin-NIT-TIG-TYL				1 (5.0)						1

a *Enterococci were isolated from BEA and BEA^E^ media*.

b *Tylosin inclusion at 11 ppm; FIRST-78% = tylosin in-feed from d 0 to d 125; LAST-75% = tylosin in-feed from d 41 to d 161; CON, control, continuous feeding of tylosin (d 0 to d 161). Fecal samples were collected on d 0, d 81, and d 160*.

c *DOX, Doxycycline; ERY, Erythromycin; LIN, Linezolid; NIT, Nitrofurantoin; Q-D, Quinupristin-dalfopristin; STR, Streptomycin; TIG, Tigecycline; TYL, Tylosin. Upper case denotes complete resistance and lower case denotes intermediate resistance*.

### Identification of Resistant Gene Determinants

Of the 153 enterococci isolates displaying intermediate resistance (n_Ery_ = 8; n_Tyl_ =7) or resistance (n_Ery_ = 139; n_Tyl_ = 144) to erythromycin or tylosin, the *erm*(B) gene was detected in 144 (Table 3) with representatives of *E. hirae, E. faecium*, and *E. villorum*. Within these isolates, six [*E. hirae* (*n* =1), and *E. faecium* (*n* = 5)] collected on day 0 were also positive for *msr*C. Nine isolates from BEA displayed intermediate resistance to either erythromycin or tylosin, but were negative for both macrolide resistance genes.

**Table 3:** Distribution of enterococci isolates from feedlot cattle feces grouped according to macrolide (*n* = 153) and tetracycline (*n*= 54) resistance genes and by cattle fed tylosin for the **FIRST-78%**, **LAST-75%**, or continuously (**CON**) during the feeding period.

**Treatment^**a**^**	**No. Positive (%)**^**b**^								
	**Macrolide**				**Tetracycline**				
	***n***	***erm*(B)**	***msr*C**	**Negative**	***n***	***tet*(L)**	***tet*(M)**	***tet*(O)**	**Negative**
FIRST-78	51	49 (96.1)	1 (2.0)	2 (3.9)	17	13 (61.9)	13 (61.9)	4 (19.0)	0 (0)
LAST-75	51	48 (94.1)	3 (5.9)	3 (5.9)	21	18 (85.7)	19 (90.5)	2 (9.5)	0 (0)
CON	51	47 (92.2)	2 (3.9)	4 (7.8)	16	10 (62.5)	10 (62.5)	5 (31.3)	1 (6.3)
Total	153	144 (94.1)	6 (3.9)	9 (5.9)	54	41 (75.9)	42 (77.8)	11 (20.4)	1 (6.3)

a *Tylosin inclusion at 11 ppm; FIRST-78% = tylosin in-feed from d 0 to d 125; LAST-75% = tylosin in-feed from d 41 to d 161; CON = control, continuous feeding of tylosin (d 0 to d 161)*.

b *Isolates pooled across all media types and sampling days*.

Within the 153 isolates screened for macrolide resistance genes, 39 displayed intermediate resistance and 15 were resistant to doxycycline. These isolates were further screened for tetracycline resistance genes, with 41 positive for both *tet*(M) and *tet*(L), and one positive for *tet*(M). Eleven isolates were positive for *tet*(O), with only one intermediate doxycycline resistant isolate being negative for all *tet* genes.

### Liver Abscesses, Animal Performance, and Carcass Traits

Although the prevalence of severe liver abscesses (A+) for the **FIRST-78%** (*P* < 0.05) was or tended to be greater **LAST-75%** (P < 0.08) than **CON** (Table 4), the overall prevalence of liver abscesses (A and A+) was similar among treatments.

**Table 4:** Growth performance, liver abscesses, and carcass traits of feedlot cattle from cattle fed tylosin for the **FIRST-78%**, **LAST-75%**, or continuously (**CON**) during the feeding period.

**Item**	**Treatments**^**a**^				***P*****-values**	
	**FIRST-78**	**LAST-75**	**CON**	**SEM**	**FIRST-78 vs. CON**	**LAST-75 vs. CON**
No. of cattle	2,525	2,526	2,525			
Growth^b^						
Initial Hip Height (m)	1.2	1.2	1.2	0.01	0.51	0.39
Initial BW (kg)	393.5	395.2	393.6	5.49	0.99	0.22
Final BW (kg)	681.0	680.0	677.5	9.25	0.25	0.40
DMI (kg/d)	11.9	11.9	11.8	0.14	0.80	0.22
ADG (kg/d)	1.8	1.8	1.7	0.03	0.25	0.69
F:G	6.7	6.8	6.8	0.07	0.23	0.70
Total liver abscesses(%)	61.0	64.2	61.9	0.4	0.81	0.53
Liver Score^c^						
0 (%)	39.0	35.9	38.1	3.64	0.81	0.53
A (%)	37.5	41.2	42.1	3.53	0.23	0.82
A+ (%)	23.5	23.0	19.8	3.92	0.05	0.08
Carcass Traits						
Carcass Weight (kg)	410.2	408.1	406.9	5.72	0.04	0.45
Dress Percentage (%)	60.2	60.0	60.1	0.1	0.20	0.61
Yield Grade						
Canada 1 (%)	21.9	21.6	20.9	3.91	0.74	0.82
Canada 2 (%)	35.9	39.2	39.0	2.11	0.11	0.92
Canada 3 (%)	42.2	39.3	40.2	5.35	0.55	0.80
Quality Grades						
Canada Prime (%)	1.0	0.8	1.2	0.25	0.62	0.25
Canada AAA (%)	69.2	64.4	66.7	2.69	0.31	0.35
Canada AA (%)	25.8	30.4	27.3	2.72	0.48	0.16
Canada A (%)	0.6	0.8	1.0	0.24	0.09	0.43
B4 (%)	3.3	3.4	3.6	1.33	0.75	0.80
Other (%)^d^	0.1	0.2	0.2	0.11	0.59	0.62

a *Tylosin inclusion at 11 ppm; FIRST-78% = tylosin in-feed from d 0 to d 125; LAST-75% = tylosin in-feed from d 41 to d 161; CON, control, continuous feeding of tylosin (d 0 to d 161)*.

b *DMI, dry matter intake; ADG, average daily gain; F:G, feed-to-gain ratio, calculated as DMI divided by ADG (live weight basis)*.

c *Liver score 0 = no abscesses (normal healthy liver); A = 1 or 2 small active abscesses/scars or up to 4 well organized abscesses >1 inch (2.5 cm) in diameter. A+ = 1 or more large active abscesses with surrounding zone of inflammation or more than 4 small/old abscesses >1 inch (2.5 cm) in diameter*.

d *Canada quality grades B2, B3, D2, D3, and E were combined into “Other” off grades category*.

There were no significant differences detected between the **FIRST-78%** or **LAST-75%** and the **CON** for any of the morbidity or mortality outcomes (Supplementary Table 2). The incidence of morbidity was <3% and the overall mortality rate ranged from 0.9 to 1.4% for all treatments.

The treatments were homogenous (*P* ≥ 0.05) at allocation with respect to average initial weight (kg) and average hip height (m) (Table 4). Growth performance of feedlot cattle did not differ (*P* > 0.05) between the **FIRST-78%** and **CON** or **LAST-75%** and **CON** for ADG or F:G (Table 4). Carcass weight was greater (absolute difference of 3.3 kg; *P* = 0.04) for cattle in the **FIRST-78%** compared to **CON** (Table 4). There was no difference detected between the **FIRST-78%** or **LAST-75%** and **CON** for dressing percentage (Table 4). Yield and quality grade also did not differ among treatments (Table 4).

## Discussion

For the purpose of this study, enterococci were chosen as the fecal indicator bacteria for assessing macrolide resistance, as *Escherichia coli* is intrinsically resistant to this antimicrobial family (30). Enterococci, notably *E. faecalis* and *E. faecium* are seen with increasing prevalence in clinical infections in humans (14). In the present study, *E. faecalis* was not detected, and *E. faecium* was only isolated from cattle upon arrival. Consistent with previous reports (16, 31, 32), there was a decrease in the diversity of enterococci over the feeding period, with *E. hirae* being the predominant species isolated from beef cattle feces, a species seldom associated with infections in humans (33). Beukers et al. (16) proposed that this shift in fecal enterococci species may arise from the transition of cattle from a forage-based to a grain-based finishing diet during the finishing period. Others have proposed that it may also be influenced by age of the host (34, 35). In the present study, cattle were transitioned from a high (40%) to low (11.5%) forage diet over the first 20 days of the feeding period. Therefore, cattle pens sampled upon allocation had less concentrate in their diets compared to those sampled on days 81 and 160 when the high concentrate diet was fed.

Tylosin was administered to cattle at the concentration approved for the prevention of liver abscesses (19). Since this study revolved around the feeding regime of tylosin, the main focus was on Ery^R^ enterococci isolated from beef cattle feces. Antimicrobial susceptibility testing of enterococci indicated that all isolates initially collected from the selective BEA^E^ were resistant to erythromycin.

A small-scale study in Southern Alberta demonstrated that although tylosin did not reduce the overall prevalence of liver abscesses, severely abscessed livers tended to be lower in cattle fed tylosin (6.7%) than in those that did not receive it (negative control; 53.3%) (36). Due to the large number of animals enrolled in this study, and the importance of tylosin in liver abscess control (37, 38), a negative control group of cattle that did not receive tylosin was not economically feasible. As in the present study, several studies have shown that in-feed tylosin increases Ery^R^ enterococci in cattle as compared to those that do not receive this antimicrobial (16, 31, 39).

The amount of Ery^R^ enterococci did not differ among treatments at any of the three sampling days. However, between the time of allocation and mid-sampling, the proportion of Ery^R^ enterococci increased and then subsequently decreased at the end of the feeding period, an observation that coincides with Beukers et al. (16). In a smaller scale study, Beukers et al. (16) compared macrolide resistance in fecal enterococci in cattle fed tylosin for the first 197 days and after withdrawal 28 days prior to slaughter. They observed a reduction in macrolide resistance, just prior to and after the removal of tylosin. Müller et al. (17) explored the intermittent use (1 week on, 2 weeks off) of tylosin compared to continuous or no tylosin and found no difference in Ery^R^ enterococci between tylosin treatment at each time point. However, these researchers did record a higher percentage of Ery^R^ enterococci with increasing days on feed between day 20 and day 118. The beneficial effect of reducing tylosin in-feed on the degree of resistance is difficult to predict because antimicrobial resistant bacteria are present in nearly all environments (40). However, shortening the duration of tylosin administered could help reduce the selection pressure that exacerbates the occurrence of antimicrobial resistance (16). In relation to the present study, to realize the impact of the removal of tylosin on the reduction in macrolide resistance, a much longer duration than 25% of the feeding period may be required.

Cattle feces are a natural vector for the transmission of bacteria and their antimicrobial resistance genes into the environment (41). Enterococci are known as antimicrobial resistance gene traffickers because they can readily transfer and acquire antimicrobial resistance genes (42). Enterococci have emerged as a major public health concern, especially vancomycin resistant *E. faecalis* and *E. faecium* which are more difficult to treat (43). Of the 176 isolates screened for antimicrobial resistance, all were susceptible to vancomycin, a result that agrees with previous studies that have suggested that cattle feces are not a major source of vancomycin-resistant enterococci (16, 44). In the present study, resistance to tylosin, erythromycin and doxycycline was most prevalent among isolated enterococci. It has been proposed that the administration of tylosin may co-select for enterococci with resistance to tetracycline, even in the absence of tetracycline use (45). Müller et al. (17) reported increased proportion of Tet^R^ enterococci in cattle feces with increasing days on feed, but found no relationship between Tet^R^ occurrence and the administration of tylosin in feed. Although tetracycline was absent in the diet, Müller et al. (17) observed an initially high proportion of Tet^R^ enterococci in cattle feces at approximately 10% on day 0, with increases between day 20 (~20%) and day 118 (~40%). These results coincide with the present study, where initially a high number of enterococci isolates with intermediate or resistant phenotypes to doxycycline (23%) was detected, with this level only increasing slightly between days 81 (34%) and 160 (31%).

Resistance of enterococci to erythromycin and tetracycline are commonly encoded by *erm*(B), *msr*C, and *tet* (L), *tet*(M), *tet*(O) resistance genes, respectively (16, 44). The resistance gene *msr*C, is universally present in all *E. faecium* (27) and was detected in all isolates of this species as well as in one *E. hirae* isolate. Other Ery^R^ genes in enterococci include *erm*(A) and *erm*(C) (27), but we did not screen for these genes as they are infrequent in enterococci isolated from beef cattle (16, 31, 46). Nine isolates were negative for both macrolide resistance genes, suggesting that these isolates contained unknown or other known macrolide resistance genes that were not screened (40, 46).

The occurrence of multiple resistance genes within a single isolate may suggest the presence of mobile genetic elements (MGE). Both *tet*(M) and *erm*(B) are known to be frequently associated with the *Tn*916 family of MGE that are common in enterococci (47). Therefore, feeding tylosin may create selective pressure for not only macrolide resistance, but also tetracycline resistance (45). Although erythromycin and tetracycline are seldom used to treat enterococcal infections, they are used to treat other bacterial infections in humans (48). If resistant enterococci serve as a reservoir of these MGE-associated antimicrobial resistance genes, they could present a public health risk (44).

Previous studies noted that liver abscesses, especially livers scored as severe (A+) result in reduced feed intake, and a lower final body weight (1). Tylosin is frequently administered in-feed throughout the entire feeding period and in the past was found to lower the prevalence of liver abscesses 40–70% (37). However, the incidence of liver abscesses in feedlot cattle has increased over time, even with the inclusion of tylosin in the diet (5). Brink (1) evaluated 12 experiments involving 566 cattle and found that on average, cattle finished at a final weight of 473.0 kg over 131 DOF had a prevalence of severe liver abscesses of 6% (Range 0–19%). Their study suggested that the risk of severe liver abscesses increase with increasing finishing weight and duration on feed. The reasons why tylosin does not completely control liver abscesses are unknown, but there are speculations it may promote the growth of opportunistic pathogens, select for resistance strains, or that its concentration in the rumen is too low to be affective against the causative bacteria (49). Although previous work has shown little evidence that exposure of *F. necrophorum* or *T. pyogenes* to tylosin promotes macrolide resistance (49–51).

In the current study, the proportion of severely abscessed (A+) livers was greater in the **FIRST-78%** (*P* < 0.05) and tended to be higher in the **LAST-75%** (*P* < 0.08) compared to the **CON**. However, the proportion of total liver abscesses was not affected when tylosin was administered for shorter durations during the feeding period. Despite the greater prevalence of severe liver abscesses with shorter duration tylosin programs, there was no difference (*P* < 0.05) between the **FIRST-78%** or **LAST-75%** and the **CON** for any of the morbidity or mortality outcomes. Overall, the mortality rate for the present study was <2% which is within the lower range (0–15%) of feedlot cattle in North America (52). The primary causes of mortality included bovine respiratory disease, lameness, metabolic disorders including bloat and acidosis. With the exception of metabolic disorders, all of the other causes of mortality were not treated with tylosin and the use of other macrolides was avoided.

Walter et al. (53) evaluated liver abscess prevalence in cattle (*n* = 3,360) fed tylosin during the first 42, first 84, last 84, and first 126 out of 162 days on feed compared to continuous or no tylosin administration. They observed a linear total decrease in abscessed and A+ livers as days of tylosin feeding increased. Cattle that were fed tylosin in the first 84 d had fewer A+ livers than cattle fed tylosin for the last 84 d, suggesting that a greatest risk of liver abscess formation and subsequent greatest efficacy if tylosin is administered early in the feeding period (53). However, in our study, the marginal difference of A+ liver score between **LAST-75%** and the **CON** suggests that there is still risk of severe liver abscess formation later in the feeding period. Similar to our study, Walter et al. (53) found a decrease in overall edible/healthy livers (score 0) with reduced tylosin administration. In the present study, the origin of the cattle was not recorded, but they were older yearling cattle. Therefore, the cattle may have had pre-existing or increased susceptibility to developing liver abscesses prior to their arrival at the feedlot. This or the fact that the feedlot diet contained a mixture of wheat and barley may account for the much higher prevalence of liver abscesses observed in our study as compared to Walter et al. (53). Using feedlot performance as a secondary indicator of animal health and welfare, no differences in mortality, ADG, F:G, hot carcass weight, marbling score or other carcass traits were observed.

## Conclusion

Few studies have investigated the effect of reduced tylosin feeding in feedlot cattle. Based on the results of our study, shortening the duration of tylosin feeding is likely to result in slightly more severe liver abscesses, but the overall impacts on morbidity and mortality, animal performance and carcass traits may be minimal in cattle fed for ~160 days. This study demonstrates that reduced feeding of tylosin either at the beginning or end of the feeding period is unlikely to significantly change the proportion of resistant enterococci in the feces at the time of slaughter. The measured levels of Ery^R^ and antimicrobial susceptibility patterns in enterococci only exhibited a modest relationship to the intermittent administration of tylosin to feedlot cattle. Additionally, *E. hirae*, was the predominant species of enterococci associated with feedlot cattle fed a high grain finishing diet, a species that is not commonly associated with infections in humans. Findings of this study support the potential for producers to reduce the administration of tylosin, a member of the macrolide class of antimicrobials that are considered important to public health. However, such practices are unlikely to reduce the amount of macrolide resistant enterococci excreted in beef cattle feces.

## Data Availability Statement

The datasets generated for this study are available on request to the corresponding author.

## Ethics Statement

All procedures involving cattle were reviewed and approved by the Feedlot Health Management Services Ltd (Okotoks, Alberta) and Lethbridge Research Center Animal Care Committees in accordance with guidelines of the Canadian Council on Animal Care (18). Written informed consent was obtained from the owners for the participation of their animals in this study.

## Author Contributions

TM, RZ, SG, CB, SH, and CK conceived the project idea and devised a plan. TD coordinated laboratory level study implementation and conduced laboratory bench work with support from HS. TM, RZ, and CN-B were involved in planning and supervising the work. AB and CK coordinated feedlot-level study implementation and collected and delivered samples to the lab. AR-G performed the statistical analysis on the bacterial data. AB compiled animal health and performance data. CB and SH helped to verify final animal health and performance data and were involved in results interpretation. TD wrote the manuscript. All authors provided critical feedback and helped shape the research, analysis and manuscript.

### Conflict of Interest

CB is part owner and managing partner of Feedlot Health Management Services Ltd. and Southern Alberta Veterinary Services Ltd. The remaining authors declare that the research was conducted in the absence of any commercial or financial relationships that could be construed as a potential conflict of interest. The reviewer MW declared a past co-authorship with one of the author TM to the handling editor.
