# Supplementary material for: Investigation of a Reduction in Tylosin on the Prevalence of Liver Abscesses and Antimicrobial Resistance in Enterococci in Feedlot Cattle
Source: Front Vet Sci. 2020 Feb 28;7:90. doi: 10.3389/fvets.2020.00090 (PMC7059211; doi:10.3389/fvets.2020.00090)
Supplement: Supplementary file 1 [file Data_Sheet_1.docx]

Supplementary Material

# Supplementary Figures and Tables

Supplementary Material should be uploaded separately on submission.

## Supplementary Figures

**Supplementary Table 1**. Antibiotics and zone diameters used for disk susceptibility testing.

| **Antibiotic** | **Supplier** | **Disk content (μg)** | **Zone diameter (mm) breakpoints^e^** | | |
| --- | --- | --- | --- | --- | --- |
|  |  |  | ***S*** | ***I*** | ***R*** |
| Ampicillin^a^ | BD | 10 | ≥17 | n/a | ≤16 |
| Doxycycline^a^ | BD | 30 | ≥16 | 13–15 | ≤12 |
| Erythromycin^a^ | BD | 15 | ≥23 | 14–22 | ≤13 |
| Gentamicin^a^ | BD | 120 | ≥10 | 7–9 | 6 |
| Levofloxacin^a^ | BD | 5 | ≥17 | 14–16 | ≤13 |
| Linezolid^a^ | BD | 30 | ≥23 | 21–22 | ≤20 |
| Nitrofurantoin^a^ | BD | 300 | ≥17 | 15–16 | ≤14 |
| Quinupristin-dalfopristin^a^ | BD | 4.5/10.5 | ≥19 | 16–18 | ≤15 |
| Streptomycin^a^ | BD | 300 | ≥10 | 7–9 | 6 |
| Tigecycline^a^ | Oxoid | 15 | ≥18 | n/a | <18 |
| Tylosin^c^ | Mast Group | 30 | ≥18 | n/a | ≤12 |
| Vancomycin^b,d^ | BD | 30 | ≥17 | 15–16 | ≤14 |

^a^ M100-S26: Performance standards for antimicrobial susceptibility testing (CLSI, 2016).

^b^ Breakpoint tables for interpretation of MICs and zone diameters (EUCAST, 2019).

^c^ (Beukers et al., 2015).

^d^ Vancomycin required 24 h of incubation, while the remaining antibiotics were assessed after 18 h.

^e^ Zone diameters (mm) are interpreted to indicate: S = susceptible, I = intermediate, R = resistant, n/a = not available

**Supplementary Table 2.** Morbidity and mortality outcomes of feedlot cattle for cattle fed tylosin for the **FIRST-78%**, **LAST-75%**, or continuously (**CON**) during the feeding period.

| Item^1^ | Treatment^2^ | | | *P* - values | |
| --- | --- | --- | --- | --- | --- |
|  | FIRST-78 | LAST-75 | CON | FIRST-78% vs CON | LAST-75% vs CON |
| Morbidity |  |  |  |  |  |
| Initial BRD Treatment (%) | 2.7 | 2.5 | 2.2 | 0.33 | 0.44 |
| Initial Bloat Treatment (%) | 0.3 | 0.2 | 0.2 | 0.71 | 0.74 |
| Initial Lameness Treatment (%) | 1.1 | 1.0 | 1.6 | 0.57 | 0.44 |
| Initial Other Treatment (%) | 0.6 | 0.7 | 1.1 | 0.16 | 0.29 |
| Chronicity (%) | 0.4 | 0.2 | 0.3 | 0.79 | 0.25 |
| Wastage (%) | 0.2 | 0.1 | 0.2 | 0.74 | 0.28 |
| Mortality |  |  |  |  |  |
| Overall Mortality (%) | 1.4 | 0.9 | 1.3 | 0.66 | 0.19 |
| BRD Mortality (%) | 0.2 | 0.1 | 0.1 | 0.34 | 1.00 |
| Lameness Mortality (%) | 0.1 | 0.0 | 0.0 | n/a | n/a^3^ |
| Metabolic Mortality (%) | 0.6 | 0.4 | 0.7 | 0.57 | 0.20 |
| Other Mortality (%) | 0.6 | 0.4 | 0.5 | 0.67 | 0.53 |

^1^ BRD = bovine respiratory disease. Lameness treatment includes arthritis, foot rot, papillomatous digital dermatitis, and lameness.

^2^ Tylosin inclusion at 11 ppm; FIRST-78% = tylosin in-feed from d 0 to d 125; LAST-75% = tylosin in-feed from d 41 to d 161; CON = control, continuous feeding of tylosin (d 0 to d 161).

^3^ n/a = not available - model would not properly converge because of the small number of events.
